# Risk factors for inadequate prenatal care use in the metropolitan area of Aracaju, Northeast Brazil

**DOI:** 10.1186/1471-2393-9-31

**Published:** 2009-07-22

**Authors:** Eleonora RO Ribeiro, Alzira Maria DN Guimarães, Heloísa Bettiol, Danilo DF Lima, Maria Luiza D Almeida, Luiz de Souza, Antônio Augusto M Silva, Ricardo Q Gurgel

**Affiliations:** 1 Department of Medicine, Federal University of Sergipe, Brazil; 2 Federal University of Sergipe, Brazil; 3 Department of Paediatrics and Child Care, Faculty of Medicine of Ribeirão Preto, University of São Paulo, Brazil; 4 Federal University of Sergipe, Brazil; 5 Postgraduate Nucleus of Medicine, Federal University of Sergipe, Brazil; 6 Department of Public Health, Federal University of Maranhão, Brazil

## Abstract

**Background:**

The aim of prenatal care is to promote good maternal and foetal health and to identify risk factors for adverse pregnancy outcomes in an attempt to promptly manage and solve them. Although high prenatal care attendance is reported in most areas in Brazil, perinatal and neonatal mortalities are disproportionally high, raising doubts about the quality and performance of the care provided. The objective of the present study was to evaluate the adequacy of prenatal care use and the risk factors involved in inadequate prenatal care utilization in the metropolitan area of Aracaju, Northeast Brazil.

**Methods:**

A survey was carried out with puerperal women who delivered singleton liveborns in all **four **maternity hospitals of Aracaju. A total of 4552 singleton liveborns were studied. The Adequacy of Prenatal Care Utilization Index, modified according to the guidelines of the Prenatal Care and Birth Humanization Programme, was applied. Socioeconomic, demographic, biological, life style and health service factors were evaluated by multiple logistic regression. Results: Prenatal care coverage in Aracaju was high (98.3%), with a mean number of 6.24 visits. Prenatal care was considered to be adequate or intensive in 66.1% of cases, while 33.9% were considered to have inadequate usage. Age < 18 to 34 years at delivery, low maternal schooling, low family income, two or more previous deliveries, maternal smoking during pregnancy, having no partner and prenatal care obtained outside Aracaju were associated with inadequate prenatal care use. In contrast, private service attendance protected from inadequate prenatal care use.

**Conclusion:**

Prenatal care coverage was high. However, a significant number of women still had inadequate prenatal care use. Socioeconomic inequalities, demographic factors and behavioural risk factors are still important factors associated with inadequate prenatal care use.

## Background

Prenatal care aims to promote good maternal and foetal health and to identify risk factors for adverse pregnancy outcomes in an attempt to promptly manage and solve them [1]. Observational studies suggest that inadequate prenatal care use is strongly associated with higher infant [2], neonatal[3,4] and foetal mortality [4] although the likelihood of these associations being causal is being questioned [5]. Evidence regarding the effectiveness of prenatal care remains equivocal [1,6-8] and good-quality evidence is scarce [9,10]. Furthermore, prenatal care has also been disappointing in reducing maternal mortality [9].

The American College of Obstetrics and Gynecology (ACOG) recommends a minimum of 11 visits, but the World Health Organization considers that, for low-risk pregnancies, a lower number of visits may be adequate if good quality care can be provided and problems are detected promptly and properly addressed [11-13]. Randomized controlled trials suggest that reduced prenatal care packages containing fewer visits can be as effective as standard models of prenatal care for low-risk women and are not associated with adverse pregnancy outcomes [10,12,14]. In developed countries the traditional model of prenatal care has been based on a large number of visits, starting as early in pregnancy as possible. This model has been exported to the developing world where persistent lack of resources may hamper prenatal care effectiveness [15].

High perinatal and maternal mortality has been reported in Brazil [16,17]. Reduced access to prenatal care in some regions prompted the Ministry of Health to institute the Prenatal and Birth Humanization Programme (Programa de Humanização do Pré-Natal e Nascimento – PHPN is the Brazilian acronym). In order to standardise procedures, this programme establishes a minimum of six visits for a full term pregnancy, with the first visit recommended to be held before 16 weeks of gestation [18,19].

Evaluation of prenatal care was proposed by Kessner in 1976 and, in 1994, Kotelchuck developed the Adequacy of Prenatal Care Utilization (APNCU), the so-called Kotelchuck Index [20,21]. Using ACOG recommendations as a reference, this index takes into account the month in which prenatal care started, the number of visits and gestational age [11,21-23].

There are some discrepancies between the Kotelchuck Index and Brazilian PHPN recommendations, mostly regarding the recommended number of visits. The usual practice is to adapt this index to Brazilian PHPN standards [7,11,24]. There is no gold standard for the evaluation of prenatal care use and different recommendations make it difficult to compare studies in different settings and conditions. More frequently studies take into account the number of visits and gestational age on commencing prenatal care [17].

Despite these controversies, a consensus about the necessity of early access to prenatal care prevails, in order to permit identification of risk in early pregnancy, to reduce maternal morbidity and the consequences for the newborn [11,22]. Thus, the objective of the present study was to evaluate the adequacy of prenatal care use and the factors associated with inadequate prenatal care use in the metropolitan area of Aracaju, Northeast Brazil, using a modified adequacy of prenatal care utilization index according to the recommendations of the Brazilian PHPN [18].

## Methods

This is a cross-sectional study of all singleton live births delivered at all four hospitals in the metropolitan area of Aracaju between March 8^th ^and July 15^th ^2005. Participating children are now being followed as part of a cohort study.

The metropolitan area of Aracaju comprises four municipalities: Aracaju (the capital city of Sergipe), Barra dos Coqueiros, Nossa Senhora do Socorro, and São Cristóvão. The latter three border Aracaju and, as the state capital has limited physical space for expansion, poor and middle income popular housing moved there.

Four maternity hospitals serve the metropolitan and are located in Aracaju. Our aim was to study one third of the number of live births, estimated at 4510 children in the previous year. No sample size calculation was performed because this was a population study in which all live births within a four month period were included since there is no seasonal variation in the number and characteristics of live births. All women who had severe diabetes, hypertension, myocardial infarction or asthma or who were unable to answer the questionnaire, or were in intensive care were excluded. During the study period 4746 women gave birth in all four hospitals of the city. Of these, 184 were excluded for the reasons mentioned above (3.88%) and a further 10 (0.2%) refused to be interviewed, leaving 4552 cases for analysis. Thus, the response rate was 95.9%.

The questionnaire comprised 114 open and closed questions applied within 24 hours of delivery. The questions were grouped into 8 themes: identification, mother's background, father's background, obstetric history, gestation, delivery, and newborn immediate health outcome. Trained research assistants held the interviews after obtaining written informed consent.

The supervisory team reviewed all questionnaires and 10% of them were applied again to ensure correct classification. Data were double entered using EPINFO 3.2.2.

The adequacy of prenatal care was evaluated using the *Adequacy of Prenatal Care Utilization Index *(APNCU) [21], modified according to the number of visits established by the Brazilian Ministry of Health (6 for ≥ 37 weeks of gestation, 5 for 33–36 weeks, 4 for 29–32 weeks, 3 for 24–28 weeks, and 2 for < 24 weeks) [18]. Adequacy of prenatal care use was classified as absent, inadequate, intermediate, adequate and intensive. Inadequate care occurred when prenatal care started after the 15^th ^week of gestation or the ratio between the actual number divided by the expected number of visits was below 50%. Intermediate, adequate or intensive care was considered to have been provided when prenatal care started before the 16^th ^week of gestation and the ratio between the actual number divided by the expected number of visits was 50% to 79%, 80% to 109% and ≥ 110%, respectively.

The chi-square test was used for univariate analysis and the level of significance was set at 0.05. Odds ratio and 95% Confidence Intervals (CI) were calculated using multiple logistic regression to evaluate independent factors related to inadequate prenatal care utilization. The model included all variables with a p value < 0.20 in univariate analysis. The dependent variable "inadequate prenatal care use" was coded as zero for "adequate and intensive" categories and as **one **for "intermediate, inadequate or no prenatal" categories. For the logistic regression analysis, the intermediate category was considered together with the absent and inadequate categories, since they involved a number of visits lower than the minimum set by the Brazilian Ministry of Health. A baseline category for each variable was established according to biological and/or previous criteria derived from the literature. The final model had 4552 observations and included information on maternal age (<18, 18 to 34 and ≥ 35 years), maternal schooling (≤ 4.5 to 8 and ≥ 9 years), family income (< 1.1 to < 3 and > 3 minimum wages), number of deliveries (≤ 1.2 to 4 and ≥ 5), marital status (cohabiting or not), municipality where prenatal care was received (Aracaju, others), type of prenatal care assistance (private, public), and smoking at least one cigarette per day during pregnancy. Maternal age was categorized as < 18 years because earlier works suggested that risk factors associated with adolescent pregnancy are only evident for women below this age [25,26]. Two variables regarding prenatal care content were also investigated, i.e., breast examination (yes, no) and receiving information about breastfeeding (yes, no) during prenatal visits. The study was approved by the Ethics Committee of the Federal University of Sergipe (number 138/2004).

## Results

Prenatal care was extensively used. Only 1.7% of patients reported not having accessed any service. When combined, the adequate and intensive categories reached 66.1%, a value above the minimum standard set by the Ministry of Health, whereas 33.9% of the women were classified as having inadequate prenatal care uptake (absent+inadequate+intermediate). Mean maternal age was 25.3 years (SD = 6.3), with a range of 13 to 49 years, and 439 mothers (9.9%) were below 18 years of age.

The mean number of visits was 6.24 and the median was 6. A low percentage of women (13.4%) started prenatal care late (≥ 16 weeks of gestational age). The median number of visits was higher for those who started care early (6) compared to those who started late (4). The median number of visits was 8 for intensive, 6 for adequate, 4 for intermediate and 5 for inadequate prenatal care use (Table 1).

**Table 1:** Number of prenatal care visits according to adequacy of prenatal care use and gestational age at initiation of prenatal care, Aracaju, Brazil, 2005.

Variable	Mean	SD	Median	IQR	n
**Adequacy of prenatal care use**					
Absent	0	0	0	0	75
Inadequate*	4.62	1.78	5	3	1216
Intermediate	3.69	0.47	4	1	245
Adequate	5.58	0.56	6	1	1103
Intensive	8.22	1.48	8	2	1909
					
**Gestational age at initiation of prenatal care (weeks)**					
< 16	6.56	2.20	6	3	3937
≥ 16	4.13	1.78	4	2	611

Total	6.24	2.30	6	3	4548

SD – standard deviation; IQR – interquartile range

* Four mothers were classified as having inadequate prenatal care use because care started ≥ 16 weeks had missing values on the number of visits.

In the unadjusted analysis, mothers aged less than 18 years were less likely (53.5%) to receive adequate or intensive prenatal care, while mothers with higher educational level (79.1%), higher family income (80.6%), fewer children (72.2%), who did not smoke during pregnancy (67.8%), who used private services (89.0%) and attended health services in Aracaju (69.1%) were more likely to reach adequate prenatal care use (Table 2). Late initiation of prenatal care was higher among women attending public services (81.1%) than among women attending private services (p < 0.001) (Table 3).

**Table 2:** Adequacy of Prenatal Care Utilization (APNCU) Index modified according to Brazilian Ministry of Health criteria, Aracaju, Brazil, 2005

Variables			Modified APNCU								
	Absent		Inadequate		Intermediate		Adequate		Intensive		Total
	n	%	n	%	n	%	n	%	n	%	n
**Maternal age (years)**											
< 18	15	3.4	167	38.0	43	9.8	90	20.5	124	33.0	439
18 to 34	53	1.4	971	26.3	182	4.9	904	24.4	1584	45.7	3694
≥ 35	5	1.6	66	21.0	10	3.2	86	27.4	147	48.1	314
											
**Maternal schooling (years)**											
≤ 4	41	4.7	345	39.5	59	6.8	192	22.0	236	27.0	873
5 to 8	26	1.6	509	32.1	119	7.5	439	27.7	491	31.0	1584
≥ 9	7	0.3	359	17.4	67	3.2	463	22.4	1173	56.7	2069
											
**Family income (minimum wages**											
< 1	26	4.8	202	37.4	42	7.8	136	25.2	134	24.8	540
1 to < 3	38	1.5	786	30.2	164	6.3	688	26.4	929	35.7	2605
>3	9	0.7	223	16.0	38	2.7	277	19.9	843	60.7	1390
											
**Number of deliveries**											
≤ 1	20	1.0	466	22.2	99	4.7	487	23.2	1031	49.0	2103
2 to 4	42	1.9	628	28.8	130	6.0	550	25.2	829	38.0	2179
≥ 5	13	4.8	126	46.7	16	5.9	66	24.4	49	18.2	270
											
**Cohabiting partner**											
Yes	40	1.1	906	24.7	195	5.3	910	24.8	1617	44.1	3668
No	35	4.0	314	35.5	50	5.7	193	21.8	292	33.0	884
											
**Municipality of prenatal care assistance**											
Aracaju	38	1.3	746	24.6	152	5.0	726	24.0	1367	45.1	3029
Others	37	2.4	474	31.1	93	6.1	377	24.8	542	35.6	1523
											
**Type of prenatal care assistance**											
Private	0	- 93	9.4	16	1.6	148	14.9	738	74.2	995	
Public	2	0.1	1124	32.3	229	6.6	954	27.4	1169	33.6	3478
											
**Maternal smoking during pregnancy**											
Yes	19	7.7	116	47.2	17	6.9	45	18.3	49	19.9	246
No	56	1.3	1104	25.6	227	5.3	1058	24.6	1860	43.2	4305

**Total**	75	1.7	1220	26.8	245	5.4	1103	24.2	1909	41.9	4552

Numbers may not add up to total because of missing values

**Table 3:** Gestational age at which prenatal care started according to type of prenatal care assistance, Aracaju, Brazil, 2005

Type of assistance	Gestational age at which prenatal care started (weeks)				
	< 16		≥ 16		Total*
	n	%	n	%	
Public	2490	81.1	581	18.9	3071
Private	968	97.1	29	2.9	997

* p < 0.001

Mothers having adequate prenatal care use were more likely to have had their breast examined (63.2% vs 49.6%) and to be informed about breastfeeding during visits (60.5% vs 46.8%) compared to those with inadequate prenatal utilization (p < 0.001) (Table 4).

**Table 4:** Breast examination and orientation about breast feeding during prenatal care according to the Modified Adequacy of Prenatal Care Use index, Aracaju, Brazil, 2005

Variable	Modified Adequacy of prenatal care use index				
	Inadequate		Adequate		Total
	n	%	n	%	
Breast Examination *					
Yes	764	49.6	1904	63.2	2668
No	776	50.4	1107	36.8	1883
					
Oriented about breastfeeding*					
Yes	718	46.8	1818	60.5	2536
No	817	53.2	1187	39.5	2004

Total	1540	100.0	3012	100.0	4552

* p < 0.001

Numbers may not add up to total because of missing values

In the adjusted model, maternal age below 18 years and between 18 and 34 years, ≤ 8 years of maternal schooling, family income < 3 minimum wages, having 2 or more other children, lone parenthood, smoking during pregnancy and accessing prenatal care outside Aracaju were all factors associated with higher inadequate prenatal service uptake. Accessing private services was a protective factor (Table 5).

**Table 5:** Adjusted analysis of factors associated with inadequacy of prenatal care use, Aracaju, SE, 2005.

	Modified APNCU (Adequacy of prenatal care use index)*					
Variable	Inadequate		Adequate			
	n	%	n	%	Adjusted OR	95%CI
**Maternal age (years)**						
< 18	225	51.3	214	48.7	4.10	2.81 – 5.99
18 to 34	1206	32.7	2488	67.3	2.03	1.50 – 2.76
≥ 35 (reference)	81	25.8	233	74.2		
						
**Maternal schooling (years)**						
≤ 4	445	51.0	428	49.0	1.72	1.40 – 2.11
5 to 8	654	41.3	930	58.7	1.43	1.21 – 1.70
≥ 9 (reference)	433	20.9	1636	79.1		
						
**Family income (minimum wages)**						
> 1	270	50.0	270	50.0	1.39	1.08 – 1.79
1 to < 3	988	37.9	1617	62.1	1.30	1.08 – 1.56
≥ 3 (reference)	270	19.4	1120	80.6		
						
**Number of deliveries**						
≤ 1 (reference)	585	27.8	1518	72.2		
2 to 4	800	36.7	1379	63.3	1.50	1.28 – 1.74
≥ 5	155	57.4	115	42.6	2.69	1.96–3.69
						
**Cohabiting partner**						
Yes	1141	31.1	2527	68.9		
No	399	45.1	485	54.9	1.65	1.39 – 1.94
						
**Municipality of prenatal care assistance**						
Aracaju (reference)	936	30.9	2093	69.1		
Others	604	39.7	919	60.3	1.18	1.02 – 1.36
						
**Type of assistance**						
Private	109	11.0	886	89.0	0.34	0.27 – 0.43
Public (reference)	1355	39.0	2123	61.0		
						
**Maternal smoking during pregnancy**						
Yes	152	61.8	94	38.2	2.25	1.69 – 2.99
No	1387	32.2	2918	67.8		

Modified APNCU index: inadequate = absent + inadequate + intermediary

adequate = adequate + intensive

OR – Odds ratio; CI – Confidence Interval

## Discussion

Perinatal assistance in the Metropolitan Area of Aracaju is characterized by a high proportion of institutional deliveries and prenatal care coverage, but there are some inadequacies that may be compromising optimal outcome. Although mean number of visits was higher than the recommended minimum and only 1.7% of the women had no prenatal care attendance, a significant proportion of women (33.9%) were still classified as having inadequate care. Even though most women started care < 16 weeks of gestational age, 13.4% still initiated prenatal care late. Socioeconomic and demographic barriers to adequate prenatal care use are still in place. Inadequate care was more likely for women < 35 years, with low schooling and family income, having 2 or more children, cohabiting with a partner, smokers, public prenatal care users and those living outside Aracaju. Inequalities regarding content of care were also evident: those with inadequate use were less likely to have had their breast examined or to have been oriented about breastfeeding.

The mean age of the women studied was 25.3 years, lower than that observed in the Southern region (26 years) in 1993 and 2004 [23,27]. Adolescent pregnancy was high, but lower than in São Luís (MA) in 1997 (29.9%) [28], although higher than in Ribeirão Preto in 1994 (17.5%) [7] and in Pelotas in 2004 (17.4%) [29]. Adolescent pregnancy is increasing in Brazil. According to the Information System on Live Births (Sistema de Informação sobre Nascidos Vivos – SINASC), 23.3% of gestations occurred in women between 10 and 19 years of age in 2001 [16]. In the present study adolescents were more likely to have inadequate prenatal care use.

Prenatal coverage was similar to that reported in more developed regions of the country (95.0% for Pelotas [23], 96.1% for Rio de Janeiro [30], 99% for Juiz de Fora [31], 96.6% for Criciúma [32], and 97.4% for Ribeirão Preto [33]) in the South and Southeast. In São Luís [28], also in the Northeastern region, a lower proportion (89.5%) of mothers attended prenatal visits. In New Zealand, prenatal care use was 99.1%, with 89.3% considered adequate [34]. In Finland, in 2007, 99.0% of patients received prenatal care, with only 1.8% attending less than the minimum number of six recommended visits [4].

There was a higher rate of inadequate care use (39%) among public care seekers compared to private care seekers (11%), but not as high as in public services of São Luís (55.4%) [11]. In both Ribeirão Preto [33] and São Luís [11] a higher rate of inadequate prenatal care was observed among public service users. Barros et al. [29] reported that these differences were due to the lower educational and income levels of public health service users. In Aracaju, even after adjustment for these two variables, public service users presented higher rates of inadequate prenatal care use, indicating that other factors may be involved.

Overall, early prenatal assistance onset (before 16 weeks) occurred for 13.4% of women, but only for 2.9% of private care users. These figures were similar to those from Pelotas (86.0%) [29] and Criciúma (83.2%)[32], but higher than those from São Luís (60.2%) [11]. The mean number of visits was similar to that reported for São Luís (6.6) [11], but lower than that reported for Pelotas (8.3) [29].

Population-based studies are important to evaluate prenatal care. However, most studies are based on secondary data, where underreporting and limitation of the variables routinely collected by health services reduce study reliability. This may lead to lack of detailed comparison and inadequate interpretation of the data [17,23]. The present study was undertaken in a metropolitan area of the Northeast region of Brazil, a less developed region of the country with very few similar studies. Through a population-based study we were able to identify relationships between several biological, socioeconomic, demographic and behavioural aspects and patterns of health care use and adequacy of prenatal care uptake. Most of our findings agree with those of several previous studies, showing important constraints of adequate prenatal care. [11,17,23,30,35]

In this study, the percentage of missing values was low. The highest proportion of missing values was for maternal age, which was not available in 105 cases (2.3%).

The proposed index of adequacy of prenatal care use cannot evaluate visit effectiveness or quality, as they are quantitative indexes. This limitation is present in the majority of studies [11,22,29,31].

Health-conscious women are likely to initiate care early and to attend visits regularly and generally they also present health-seeking behaviors, a fact that might have provoked selection bias. No information about non-responders was collected and thus it was not possible to assess if non-responders differed from responders on any important variable. However, since the response rate was very high, it is unlikely that this caused selection bias.

The modified APNCU classified all women who initiated prenatal care after 16 weeks as receiving inadequate care regardless of the number of visits made. This is a limitation of this classification because the Brazilian Ministry of Health recommends a minimum of six visits for women with uncomplicated pregnancies. However, few women were classified as having inadequate care based on late attendance only, and most of them also had received fewer than the recommended number of visits. At least three studies have introduced changes in the APNCU [7,11,30], with that by Leal et al. [30] being the one most similar to ours.

## Conclusion

In conclusion, prenatal care coverage was high. However a significant number of women still have inadequate prenatal care use. As others have shown [33,36], our findings point to the fact that socioeconomic inequalities, demographic factors and behavioural risk factors are still important factors associated with inadequate prenatal care use in developing countries.

## Competing interests

The authors declare that they have no competing interests.

## Authors' contributions

ERAO, AMDGN and DDFL: conception and design of the study, data collection and analysis, manuscript writing and revision. RQG and MLDA: data collection and analysis, and paper writing and revision. AAMS: analysis and interpretation of data, and paper revision. LS: statistical analysis and revision of the manuscript. HB: data collection and analysis, and manuscript writing and revision. All authors read and approved the final manuscript.

## Pre-publication history

The pre-publication history for this paper can be accessed here:


